# Hydrogenase and Nitrogenase: Key Catalysts in Biohydrogen Production

**DOI:** 10.3390/molecules28031392

**Published:** 2023-02-01

**Authors:** Jinsong Xuan, Lingling He, Wen Wen, Yingang Feng

**Affiliations:** 1 Department of Bioscience and Bioengineering, School of Chemistry and Biological Engineering, University of Science and Technology Beijing, 30 Xueyuan Road, Beijing 100083, China; 2 CAS Key Laboratory of Biofuels, Shandong Provincial Key Laboratory of Synthetic Biology, Shandong Engineering Laboratory of Single Cell Oil, Qingdao Institute of Bioenergy and Bioprocess Technology, Chinese Academy of Sciences, 189 Songling Road, Qingdao 266101, China; 3 Shandong Energy Institute, 189 Songling Road, Qingdao 266101, China; 4 Qingdao New Energy Shandong Laboratory, 189 Songling Road, Qingdao 266101, China; 5 University of Chinese Academy of Sciences, Beijing 100049, China

**Keywords:** biohydrogen production, hydrogenases, nitrogenases, enzyme engineering, biocatalyst

## Abstract

Hydrogen with high energy content is considered to be a promising alternative clean energy source. Biohydrogen production through microbes provides a renewable and immense hydrogen supply by utilizing raw materials such as inexhaustible natural sunlight, water, and even organic waste, which is supposed to solve the two problems of “energy supply and environment protection” at the same time. Hydrogenases and nitrogenases are two classes of key enzymes involved in biohydrogen production and can be applied under different biological conditions. Both the research on enzymatic catalytic mechanisms and the innovations of enzymatic techniques are important and necessary for the application of biohydrogen production. In this review, we introduce the enzymatic structures related to biohydrogen production, summarize recent enzymatic and genetic engineering works to enhance hydrogen production, and describe the chemical efforts of novel synthetic artificial enzymes inspired by the two biocatalysts. Continual studies on the two types of enzymes in the future will further improve the efficiency of biohydrogen production and contribute to the economic feasibility of biohydrogen as an energy source.

## 1. Introduction

With the development of human society and the increased global energy demands, discovering alternative energy carriers and developing sustainable and eco-friendly energy-producing technologies are needed to free our social progress from the heavy dependence on natural and nonrenewable fossil fuels.

Hydrogen is the third most abundant element on earth. Hydrogen energy is a kind of high specific energy (120–142 MJ/kg compared to 44 MJ/kg for gasoline), with zero carbon-emission and water (H_2_O) being the only product [1,2]. At present, hydrogen is expected to be the most promising future alternative and clean energy source and has also been utilized in some industrial areas such as chemical synthesis, steel processing, and fertilizer production. Up to now, there are various technologies for hydrogen production. Most of the hydrogen for industrial use is produced from fossil resources by conventional methods under high-temperature and high-pressure conditions [3,4]. Electrolysis can also be used to generate hydrogen from water with (photo)-electrochemical systems [5,6]. The former method is non-renewable and releases greenhouse gases, while the latter may be costly even though it is practical [7]. All these limitations promote researchers to develop more efficient, sustainable, and environmentally harmless technologies for hydrogen production.

Biohydrogen production (BHP) systems use microorganisms to generate hydrogen from various sources. BHP depends on a series of biological processes in algae, microalgae, or bacteria, such as fermentative metabolism and N_2_ fixation [8,9,10]. BHP systems provide attractive opportunities for global energy supply in a green and renewable way. Many efforts have been made to identify novel bacterial species for hydrogen production, to perform strain improvement for the enhancement of hydrogen production, to understand molecular catalytic mechanisms of the involved enzymes including hydrogenase and nitrogenase, and to genetically engineer these biocatalysts for optimizing biohydrogen production [11,12,13].

In BHP systems, two types of metal enzymes, i.e., hydrogenase and nitrogenase, are the key catalysts with different mechanisms and reactions for hydrogen production [14,15]. Hydrogenase catalyzes the reversible reaction of hydrogen formation from protons or hydrogen oxidation to protons. Differently, nitrogenase is an irreversible catalyst and it can reduce nitrogen to ammonia and generate hydrogen as a byproduct under anaerobic and nitrogen-deficient conditions.

A good understanding of the catalytic mechanisms of both these enzymes and intensive research on technical innovations are important and necessary for the development and applications of biohydrogen production. The structures, catalytic mechanisms, and physiological functions of these enzymes have been extensively studied for many years and many advances have been achieved in recent years. However, because these enzymes have complex structures involving intricate metal clusters and cofactors, it is challenging to obtain a thorough understanding of the hydrogen production mechanism and the rational design of engineered microorganisms with higher BHP productivity. In this review, we focus on hydrogenases and nitrogenases involved in biological hydrogen production, including their structures and properties, recent engineering research on these enzymes, and the development of synthetic artificial enzyme-mimic catalysts. This knowledge will help researchers to develop new advanced BHP systems and solve energy and environmental problems in the future.

## 2. Biohydrogen Production (BHP) Systems

For decades, algae and bacteria have been known to have H_2_-centered metabolic pathways which efficiently generate hydrogen as a metabolite or a by-product. These bioprocesses give a bright prospect for a future abundant sustainable energy supply, emerging as the research area of BHP. BHP systems utilize these organisms and can incorporate modern genetic engineering methods to improve efficiency, which provides promising techniques for producing biohydrogen from inexhaustible sources—water and solar energy [16].

Microorganisms produce hydrogen via biological routes categorized as biophotosynthetic activity and fermentative metabolism. Each of them can happen either in a light-dependent process or in a light-independent process.

### 2.1. Biophotolysis

Cyanobacteria and green algae can be used to generate biohydrogen via a water-splitting process with light energy [10]:

2H_2_O + light energy → 2H_2_ + O_2_ (1)

This reaction is very simple and can be mediated by [NiFe] and [FeFe] hydrogenases and nitrogenase synthesized in specific strains. Both photosystem I (PSI) and photosystem II (PSII) are involved in this process. Light energy is harvested in PSII and water is split into O_2_ and electrons. These electrons are then activated in PSI and used for hydrogen production [3]. Direct biophotolysis can transfer light energy to hydrogen and is considered to be an attractive biohydrogen production method, but its natural hydrogen production rate is very low because of the high reaction free energy (+237 kJ/mol hydrogen) and the suppression effects of the byproduct O_2_ on involved enzymes. Genetic engineering strategies have been used to reduce the O_2_ sensitivity and increase the hydrogen production yield [17,18].

During photosynthesis, solar energy can also be transformed into chemical energy accumulated as starch or glycogen, then these compounds can be further metabolized to generate hydrogen by an anaerobic fermentation process in either dark or light conditions. In this indirect biophotolysis process, the production of O_2_ and H_2_ is separated into two chemical reactions [19].

6H_2_O + 6CO_2_ + light energy → C_6_H_12_O_6_ + 6O_2_ (2)

C_6_H_12_O_6_ + 2H_2_O → 4H_2_ + 2CH_3_COOH + 2CO_2_ (3)

This spatial separation in heterocysts has the advantage to maintain a low O_2_ concentration during hydrogen production [20]. Indirect biophotolysis has been applied for hydrogen production using green algae and cyanobacteria. These species were first cultivated to fix CO_2_ via photosynthesis, then were transferred in a sulfur deprivation condition which can slow down O_2_-induced nitrogenase/hydrogenase inactivation and allow more hydrogen production [20,21,22].

### 2.2. Fermentation

Organics such as acetate and lactate can be used as an energy source for biohydrogen fermentative production [18]. In photo-fermentation, purple non-sulfur (PNS) bacteria can convert light energy into ATP molecules which are utilized by nitrogenase to generate hydrogen [23]. During this process, water is not split and O_2_ is not produced, so the O_2_ inhibition of direct photolysis automatically disappears in photo-fermentation. Photo-fermentation can be theoretically applied to various types of organic substrates including organic wastes, but the drawbacks of low light-conversion efficiency, considerable ATP requirement, and expensive photo-bioreactor discourage its further applications [24]. The reaction catalyzed by nitrogenases depends on the type of nitrogenase [3]:

Mo-nitrogenase: N_2_ + 8H^+^ + 8e^−^ + 16ATP → 2NH_3_ + H_2_ + 16ADP + 16Pi (4)

Absence of nitrogen: 2H^+^ + 2e^−^ + 4ATP → H_2_ + 4ADP + 4Pi (5)

V-nitrogenase: N_2_ + 12H^+^ + 12e^−^ + 24ATP → 2NH_3_ + 3H_2_ + 24ADP + 24Pi (6)

Fe-nitrogenase: N_2_ + 24H^+^ + 24e^−^ + 48ATP → 2NH_3_ + 9H_2_ + 48ADP + 48Pi (7)

These equations show that hydrogen production by nitrogenases is energetically highly expensive. The energy demand needs to be fulfilled by an energy-generating process such as photosynthesis.

In dark fermentative biohydrogen production (DFBHP), hydrogen is generated from carbohydrates without the direct input of light energy, so it is possible to produce hydrogen night and day and no photo-bioreactor is required [25]. Besides these advantages, DFBHP is also considered to be a promising process for its high hydrogen yield, fast hydrogen generation rate, and utilization of diverse fast-growing bacteria [17,26]. In addition, organic waste materials and renewable lignocellulosic biomass sources are found to be potential substrates for dark fermentation, which is remarkable for the simultaneous realization of clean energy supply and environment protection [27,28]. By complete conversion, 1 mol of glucose gives 12 mol of hydrogen molecules:

C_6_H_12_O_6_ + 6H_2_O → 12H_2_ + 6CO_2_ (8)

However, considering the bacterial growth and metabolism, the hydrogen yield is restricted to a maximum of 4 mol of H_2_ per mol of glucose (Thauer limit) [29]. The reaction with acetic acids as the metabolism byproduct is

C_6_H_12_O_6_ + 2H_2_O → 4H_2_ + 2CH_3_COOH + 2CO_2_ (9)

Other constraints of the DFBHP process include pH fluctuation, the accumulation of various metabolites such as volatile fatty acids, and inhibitory byproducts such as phenolics and furan derivatives [30]. To make DFBHP more practical for industrial applications, various process improvement strategies have been developed, such as efficient anaerobic bacteria screening, bacteria immobilization, bacteria co-culturing, and strain metabolic pathways regulation [31,32]. Some studies showed that engineered hyperthermophilic anaerobe and artificial microbial consortia can produce hydrogen beyond the Thauer limit, providing new strategies to improve hydrogen productivity and yield [33,34].

## 3. Key Enzymes Involved in Biohydrogen Production and Their Structural Characteristics

Hydrogenases have been found in all domains of life and they can reversibly convert hydrogen into protons and electrons [14]. Nitrogenases are irreversible catalysts existing in diverse bacteria and archaea to generate hydrogen as a byproduct of biological N_2_ fixation (BNF) [35]. Both are key catalysts involved in biohydrogen production. Extensive research has been conducted to elucidate the structure and catalytic mechanisms of these enzymes, to manipulate the regulation of the enzyme-encoding gene expression, and to engineer the enzymes for property improvement and process optimization.

**Figure 1:**
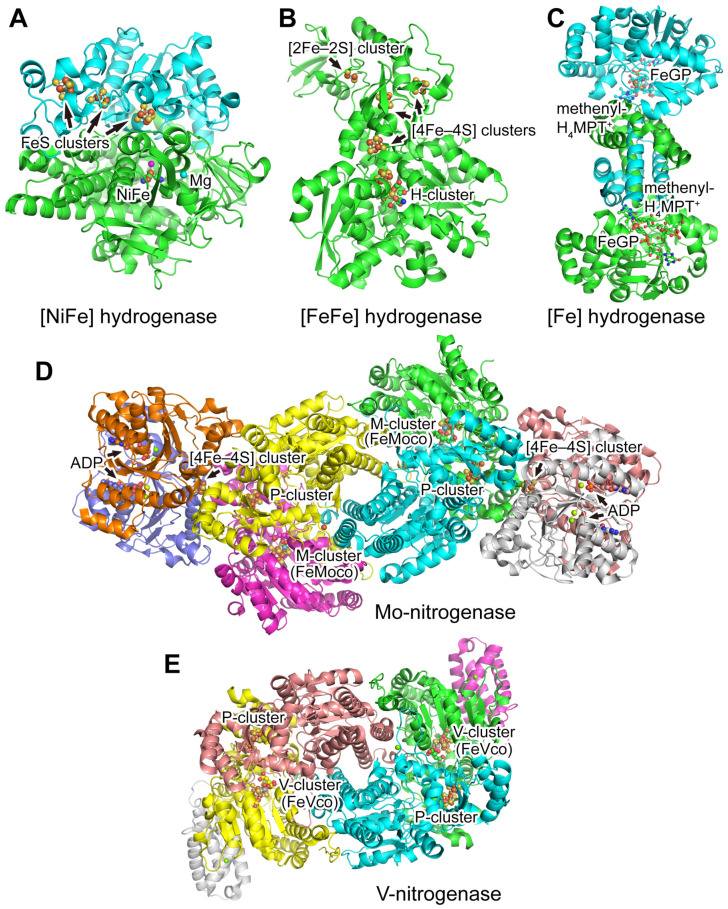
Structures of hydrogenases and nitrogenases. The proteins are shown as cartoons and different chains are colored differently. The metal clusters as well as other cofactors are labeled and shown as balls and sticks with the color codes according to the elements: C, the same color as the protein chain; N, blue; O, red; Fe, brown; Ni, magenta; Mo, dark cyan; V, dark gray; S, yellow; H, light gray; Mg, cyan; F, light cyan. (**A**) Structure of [NiFe] hydrogenase from *Desulfovibrio vulgaris* Miyazaki F (PDB 4U9H) [41]. (**B**) Structure of [FeFe]-hydrogenase from *Clostridium pasteurianum* (PDB 3C8Y) [42]. (**C**) Structure of [Fe]-hydrogenase from *Methanocaldococcus jannaschii* (PDB 3DAG) [38]. (**D**) Structure of Mo-nitrogenase from *Azotobacter vinelandii* (PDB 7UTA) [43,44]. (**E**) Structure of V-nitrogenase from *Azotobacter vinelandii* (PDB 5N6Y) [39].

**Figure 2:**
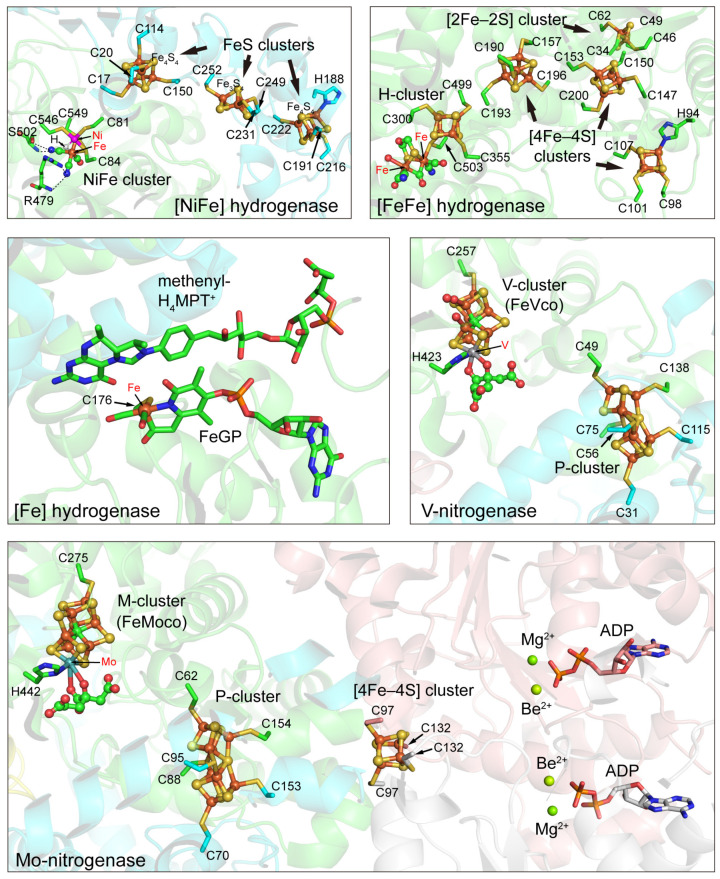
Cofactors and metal clusters in the structures of various hydrogenases and nitrogenases. The coordinating residues in proteins are shown as sticks. The PDB accession numbers, element color codes, and references of the structures are the same as those in Figure 1.

Both hydrogenase and nitrogenase are metal enzymes and most of them contain complex metal clusters and various cofactors. Great efforts have been made to elucidate the structure and mechanism of the enzymes and their ligands (Figure 1 and Figure 2). Both hydrogenase and nitrogenase can be classified into several groups (Table 1) with different structures and cofactors as shown below.

**Table 1:** Different types of hydrogenases and nitrogenases.

Enzymes	Species	Protein Components	Cofactor	Fe–S Clusters	Active Site	Reference
[NiFe] hydrogenase	*Desulfovibrio gigas*	Two subunits	[NiFe] cluster	Two [4Fe–4S] and one [3Fe–4S]	[NiFe] cluster	[14]
[FeFe] hydrogenase	*Clostridium pasteurianum*	Single subunit	H-cluster	Three [4Fe–4S] and one [2Fe–2S]	H-cluster	[36]
[Fe] hydrogenase	*Methanothermobacter marburgenis*	Homodimer	FeGP	None	Fe (II) site of FeGP	[37,38]
Mo-nitrogenase	*Rhodopseudomonas palustris*	Dimer of heterodimer	M-cluster (FeMoco)	P-cluster and one [4Fe–4S]	FeMoco	[15]
V-nitrogenase	*Azotobacter chroococcum/Azotobacter vinelandii*	Dimer of heterodimer	V-cluster (FeVco)	P-cluster and one [4Fe–4S]	FeVco	[39]
Fe-nitrogenase	*Rhodobacter capsulatus*	Dimer of heterodimer	Fe-cluster (FeFeco)	P-cluster and one [4Fe–4S]	FeFeco (proposed)	[40]

### 3.1. Hydrogenases

Hydrogenase was first discovered in bacteria in 1931 by Stephenson and Stickland [45]. Hydrogenases are a class of metal enzymes and can be divided into [NiFe] hydrogenases, [FeFe] hydrogenases, and [Fe] hydrogenases according to the different metal ions contained in the active center [46]. Although different hydrogenase classes are similar in cofactor structure and function, they are not phylogenetically related [47].

#### 3.1.1. [NiFe] Hydrogenases

[NiFe] hydrogenases are the most studied class of hydrogenases and are found in both bacteria and archaea. They act as bidirectional enzymes that can both produce and consume hydrogen, but usually, they are more active when oxidizing hydrogen. More than one kind of [NiFe] hydrogenase exists in almost all anaerobic bacteria and archaea, implying the importance of hydrogen metabolism.

[NiFe] hydrogenases can be further subdivided into five different groups according to the molecular phylogeny: uptake [NiFe] hydrogenase (group 1), cyanobacterial uptake [NiFe] hydrogenase and sensory hydrogenase (group 2), bidirectional heteromultimeric cytoplasmic [NiFe] hydrogenase (group 3), energy-converting [NiFe] hydrogenase (group 4), and actinobacterial hydrogenase (AH) (group 5) [14]. Bacterial hydrogenases mainly belong to groups 1 and 2, while group 5 hydrogenases, discovered in 2013, are widespread in soil-living actinobacteria [48]. Many members of group 3 and the majority members of group 4 are found in archaea, while genome sequencing has also discovered the presence of bacterial hydrogenases belonging to groups 3 and 4 [46]. The reactivity and function of these groups vary greatly, but they all depend on a heterobimetallic [NiFe] cofactor. In the hyperthermophilic archaea *Pyrococcus furiosus*, three [NiFe] hydrogenases were discovered. Two of them are cytoplasmic hydrogenases involved in hydrogen recycling and one membrane-bound hydrogenase is responsible for hydrogen formation with an extremely high ratio (250:1) of hydrogen evolution activity to hydrogen uptake activity [49,50]. Elevated temperature can cause an increase in hydrogen production according to the Gibbs free energy relationship of a 20% entropy enhancement by a temperature increase from 37 °C to 100 °C [51]. Hyperthermophilic archaea such as *P. furiosus*, *Thermococcus onnurineus* NA1, and *Thermococcus kodakarensis* KOD1 are prolific hydrogen producers [51].

The first crystal structure of [NiFe] hydrogenase from *Desulfovibrio gigas* was published in 1995 and more crystal structures of [NiFe] hydrogenases from other microorganisms were subsequently reported [52]. The standard [NiFe] hydrogenase consists of a large subunit (62.5 kDa) containing the bimetallic [NiFe] cluster and a small subunit (28.8 kDa) containing several iron–sulfur clusters (Figure 1A) [52].

**Figure 3:**
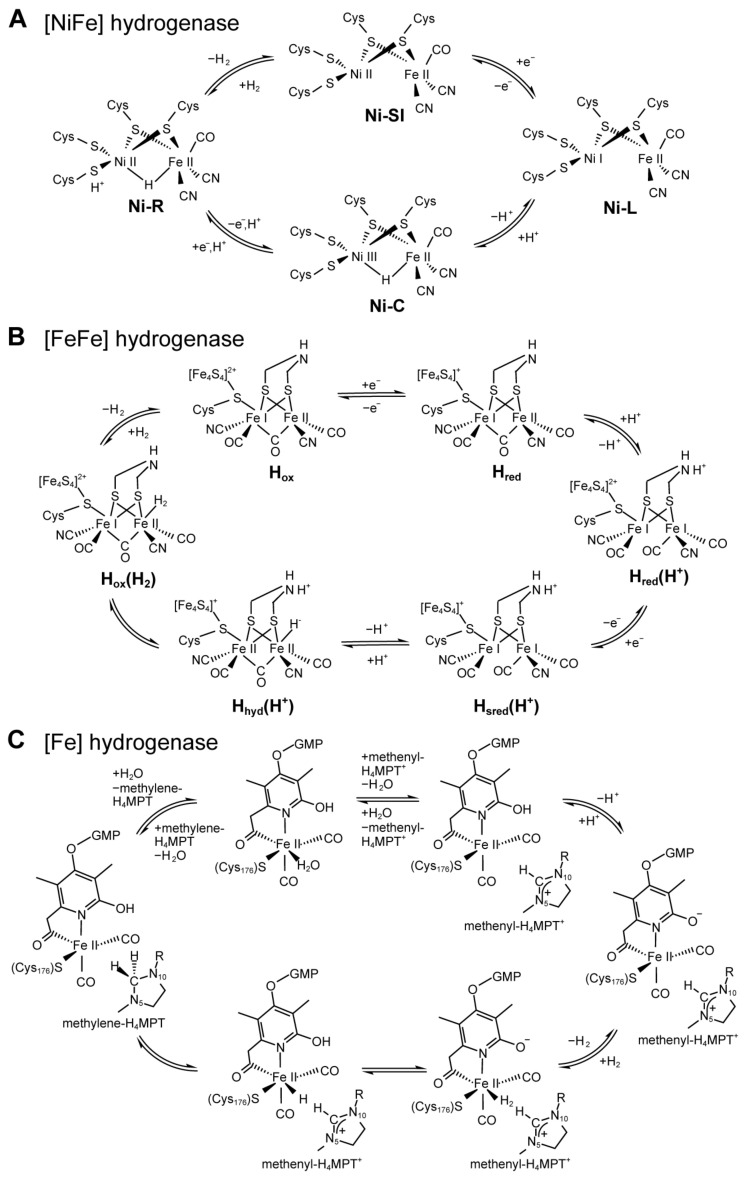
Catalytic mechanism of hydrogenases. (**A**) Catalytic cycle of [NiFe] hydrogenase [47]. (**B**) Catalytic cycle of [FeFe] hydrogenase [56]. (**C**) Catalytic cycle of [Fe] hydrogenase [57].

The bimetallic [NiFe] cluster in the large subunit is considered to be the catalytic center deeply buried inside hydrogenase, and the active site structures of all [NiFe] hydrogenases are very similar [53]. In the unique [NiFe] cluster, the Ni ion is coordinated with four cysteine residues by the S atoms, and the Fe ion is bound to the Ni ion by two bridging thiol ligands and also coordinated by non-proteinous ligands including one CO and two CN (Figure 2). In addition, a third bridging ligand exists between Ni and Fe, and it is a hydride ligand whose state changes continuously in the redox of hydrogenase. Ni appears to be in a pentacoordinate tetragonal cone configuration and Fe is in a hexacoordinated deformed octahedral configuration [14]. Substrates go through hydrophobic gas channels from the active site and the molecular surface. During the catalytic process of [NiFe] hydrogenase, the Fe(II) ion is non-redox active and maintains a low-spin state, whereas Ni changes its oxidation state: Ni(III) or Ni(II) [54]. The X-ray crystal structure of the reduced form of [NiFe] hydrogenase reveals that the third monatomic bridge at the NiFe site of the oxidized enzyme keeps this site unoccupied in the reduced form, suggesting that the movement of the monatomic sulfur bridge is necessary for the enzyme to perform its catalytic action [55]. Many mechanistic studies have discovered that different [NiFe] hydrogenases share similar mechanisms. In the inactive oxidized form of [NiFe] hydrogenase, the third bridging ligand is OH^−^, and it is removed once the enzyme is activated. Then, hydrogen binds to the [NiFe] cofactor and is cleaved heterolytically, and the Ni-R state and Ni-C state are yielded by electron reduction one by one (Figure 3A) [41,47].

The small subunit of the standard [NiFe] hydrogenase contains the proximal and distal [4Fe–4S] clusters and the medial [3Fe–4S] cluster for electron transfer between the active [NiFe] site in the center and the surface of the protein [58]. The crystal structure studies revealed that the proximal [4Fe–4S] cluster exists in two different forms which were supposed to be related to the feature of O_2_ sensitivity. Both the medial and the distal iron–sulfur clusters are located at the C-terminal of the small subunit and are well conserved in [NiFe] hydrogenases. The distal [4Fe–4S] is considered to be the final part of the electron transfer chain.

[NiFeSe] hydrogenase belongs to a subclass of [NiFe] hydrogenases and has one terminal cysteine residue replaced by a selenocysteine encoded by TGA. Structural studies found many structural differences between [NiFeSe] hydrogenase and [NiFe] hydrogenase, such as the active site, the medial FeS cluster, and metal sites at the C-terminal region of the large subunit [14]. The difference in the active site influences the properties of [NiFeSe] hydrogenase greatly: [NiFeSe] hydrogenase becomes active immediately upon reduction compared to the slow reductive activation process of standard [NiFe] hydrogenase, therefore catalyzing faster and showing a higher hydrogen production activity [59].

#### 3.1.2. [FeFe] Hydrogenases

[FeFe] hydrogenases are also bidirectional enzymes, but their specific hydrogen production rates are more than 100 times faster than those of [NiFe] hydrogenases. Unlike [NiFe] hydrogenases which tend to function as uptake hydrogenases, [FeFe] hydrogenases are usually involved in hydrogen production, so they are thought to be logical choices to increase biohydrogen production [17]. [FeFe] hydrogenases are commonly found in anaerobic prokaryotes, some anaerobic eukaryotes, and some green algae. Most well-studied [FeFe] hydrogenases are from green algae, *Clostridium*, *Desulfovibrio desulfurican*, and *Thermotoga maritima* [60,61].

Unlike [NiFe] hydrogenases consisting of at least two subunits, [FeFe] hydrogenases are often encoded in one polypeptide chain which may be monomeric or form homo-oligomers (Figure 1B). The smallest [FeFe] hydrogenases are found in green algae and consist of the catalytically active H-cluster as the only metal cluster [14].

The structural research revealed that the overall structure of the core domain which contains the H-cluster is highly conserved in different [FeFe] hydrogenases. The H-cluster is a hexanuclear iron complex buried at the center of the [FeFe] hydrogenase just like the active site of [NiFe] hydrogenase, suggesting the existence of substrate pathways in the enzyme. The H-cluster is composed of two sub-clusters: a cubane [4Fe–4S] sub-cluster responsible for electron transfer and a [2Fe–2S] sub-cluster representing the actual active center, linked by a cysteine sidechain (Figure 2) [36]. In the [2Fe–2S] sub-cluster, each metal is coordinated by one CO and one CN ligand. The CN ligands regulate the redox potential of the H-cluster by increasing the electron density of iron ions [62]. The distal Fe is the binding site of H_2_ and two sub-clusters are found to change their oxidation states which can store the two electrons for the reversible reaction of hydrogen formation from protons or hydrogen oxidation to protons in the catalytic cycle (Figure 3B). Protonation-coupled electronic rearrangement may be valid for both bacterial and eukaryotic [FeFe] hydrogenases because of the highly conserved H-cluster structures [56].

According to the crystal structures, three hydrophobic gas-access channels may exist for the substrate (H_2_) or inhibitors (CO and O_2_) to go through in [FeFe] hydrogenase. These pathways are much shorter than that of [NiFe] hydrogenase, implying the higher turnover rate of [FeFe] hydrogenase. Studies on these channels may provide new possibilities to obtain O_2_-tolerant hydrogenases.

#### 3.1.3. [Fe] Hydrogenases

[Fe] hydrogenase was first discovered in *Methanothermobacter marburgenis* in 1990 [63]. [Fe] hydrogenases catalyze the reduction of CO_2_ with H_2_ to CH_4_ and are only found in methanogenic archaea under dark conditions. They are also known as “H_2_-forming methylenetetrahydromethanopterin (methylene-H_4_MPT) dehydrogenase” (Hmd) because of their ability to catalyze the reversible reduction of methyl-H_4_MPT^+^ with H_2_ to methylene-H_4_MPT [64].

[Fe] hydrogenase does not contain Fe–S clusters or nickel but contains a unique iron-guanosylpyridol cofactor (FeGP) in which the iron ion does not change the oxidation state during catalysis (Figure 1 and Figure 2) [47]. Recent studies have found that the low-spin iron ion in the active center is Fe (II) which can act as a Lewis base for H_2_ heterolytic cleavage in the presence of MPT^+^, and the catalytic reaction is shown in Figure 3C. The low-spin Fe (II) is coordinated by two *cis*-CO ligands, a cysteine-derived thiol ligand, and a bidentate pyridinol acyl ligand [37]. The vacant position *trans* to the acyl ligand is considered to be the H_2_ binding site. The 2-hydroxy group of the pyridinol ligand is deprotonated and provides the 2-O^−^ group as an intramolecular proton acceptor to trigger H_2_ activation during catalysis [65].

Crystal structural studies show that [Fe] hydrogenases exist in open and closed conformations, respectively [66]. The substrate methyl-H_4_MPT^+^ binding can change the enzyme structural conformation from open to closed. Cys176 of [Fe] hydrogenases were identified to bind the Fe of FeGP and are essential for enzyme activity. The Fe of the activated FeGP cofactor binds a hydrogen molecule and cleaves it. The requirement of a second substrate methyl-H_4_MPT^+^ makes the catalytic mechanism of [Fe] hydrogenase different from that of [NiFe] and [FeFe] hydrogenases.

### 3.2. Nitrogenases

Nitrogenases are found in both archaea and bacteria. They are essential for the nitrogen cycle on Earth. Nitrogenases reduce N_2_ to ammonia and produce hydrogen as a byproduct. Nitrogenases contain two components, dinitrogenase and dinitrogenase reductase (Fe protein), with unique metal centers (Figure 1 and Figure 2). Three types of nitrogenases have been identified, including Mo-nitrogenase, V-nitrogenase, and Fe-nitrogenase, and their dinitrogenases have a unique metal center containing molybdenum, vanadium, and iron, respectively [67]. Therefore, the dinitrogenases are also named MoFe protein, VFe protein, and FeFe protein, and the clusters are named M-cluster, V-cluster, and Fe-cluster, respectively. These clusters are the cofactors containing the catalytic site and are also named FeMo cofactor (FeMoco), FeV cofactor (FeVco), and FeFe cofactor (FeFeco).

Most nitrogenase-containing microorganisms encode Mo-nitrogenase only, while some N_2_-fixing microorganisms encode additional V-nitrogenase and Fe-nitrogenase. *Azotobacter vinelandii*, *Methanosarcina acetivorans*, and *Rhodopseudomonas palustris* encode all three nitrogenases, while *Rhodobacter* species only encode Mo- and Fe-only nitrogenases, and *Anabaena* species encode Mo- and V-nitrogenases [35]. The three nitrogenase systems in one microorganism have a certain degree of overlap because the gene clusters for V-nitrogenase and Fe-nitrogenase do not contain the entire set of necessary nitrogenase-biosynthesis genes [35].

MoFe protein is an α_2_β_2_ heterotetramer. The subunits α and β encoded by the *nifD* and *nifK* genes, respectively, have similar folds, suggesting an evolutionary relationship between them. MoFe protein binds four [4Fe–4S] clusters consisting of two FeMo cofactors serving as substrate reduction sites and two P-clusters for initially accepting electrons from Fe protein [15]. Each of the α and β subunits contain three globular consecutive Rossmann-fold domains and the C-terminal loops from the β sheet of each Rossmann domain interact with the FeMo cofactor and the P-cluster [68]. Rossmann domains are phylogenetically related suggesting that the complex nitrogen-fixing systems can be traced back to a single domain protein functioning as a ligand for an iron–sulfur cluster [15]. The composition of the FeMo cofactor is [Mo–7Fe–9S–C]:homocitrate and its first structural model was available in 1992 [69]. Mo can support two-electron transfer reactions or oxygen atom transfer in biology. Mo^3+^ is discovered in the FeMo cofactor and this single Mo center is the site for the catalytic reduction of dinitrogen to ammonia [70]. In V-nitrogenase, Mo^3+^ is replaced by V^3+^ with the same binding geometry and the composition of the FeV cofactor is [V–7Fe–8S–C]:homocitrate [39]. The structures of the FeV cofactor and its Mo-containing analog are similar except one of the μ_2_-sulfide ligands is replaced with a carbonate ligand. The catalytic activity of vanadium nitrogenase only shows about one-third of that of molybdenum nitrogenase [15]. The structure model of the FeFe cofactor was proposed based on available data because of the lack of crystal structure of Fe-nitrogenase. Its catalytic mechanism and the protein environment around the FeFe cofactor were reported to be similar to that of V-nitrogenase and Fe-nitrogenase [40].

The architecture of the P-cluster is highly conserved for an electron relay from the Fe protein to the active site FeMo cofactor and the P-cluster is formed during nitrogenase maturation [71]. Structural analysis shows that the P-cluster has two different conformations, i.e., P^N^-form and P^Ox^-form, and this feature of structural flexibility may be linked to the electron release and ATP hydrolysis [72].

Fe protein encoded by the *nifH* gene is a dimer with two highly conserved subunits coordinating a [4Fe–4S] cluster. Bioinformatics studies showed that the Fe protein has a nucleotide-binding function and is a member of the dimeric P-loop containing ATPases and GTPases [73]. Further crystal structure research discovered conserved structural feathers such as Cys97 and Cys132 from each subunit coordinating the [4Fe–4S] cluster and the nucleotide-binding motifs located at the dimer interface, suggesting that the electron transfer and ATP hydrolysis are indirectly coupled by allosteric changes [73,74].

## 4. Bioengineering Approaches for Hydrogen Production

A deep understanding of the hydrogen metabolism and regulation is necessary to conduct genetic engineering for enhancing hydrogen production. Hydrogenases are widespread in microbes and they are involved in various metabolic pathways such as methane formation pathway, nitrogen fixation with nitrogenase-hydrogenase co-regulation, remediation of toxic heavy metals, and the virulence of pathogenic bacteria and parasites [46]. Different from the hydrogenases involved in many different metabolic pathways, nitrogenases are responsible for converting dinitrogen into ammonia in biological nitrogen fixation, providing a nitrogen source for microorganisms [75].

Extensive research on molecular mechanisms and physiological functions of hydrogenases and nitrogenases has paved new ways for enzyme engineering to improve biohydrogen production efficiency. In this section, six different bioengineering strategies will be discussed.

### 4.1. Improvement of O_2_ Tolerance

O_2_ is an important regulating factor in anaerobic hydrogen production. Both nitrogenases and hydrogenases are sensitive to O_2_. Nitrogenases must be manipulated under anaerobic conditions and their mechanisms of O_2_ inactivation are possibly oxidative damage of metalloclusters [15]. A total of 5–10% of cells in many filamentous cyanobacteria are specially differentiated cells which are called heterocysts, and they can isolate nitrogenases and provide a microaerobic environment for hydrogen production from protons [76].

Hydrogenases have different O_2_ sensitivities and need to either be protected from or tolerate the presence of O_2_ during photosynthetic hydrogen production. A comparison of the enzymes is shown in Table 2. Many efforts have been made to understand the O_2_ tolerance in hydrogenases and to obtain O_2_-tolerant enzymes by engineering.

A few O_2_-tolerant hydrogenases exist in nature. Natural O_2_-tolerant [FeFe] hydrogenase was found in *Clostridium bjerinckii* SM10 (CbA5H) [77]. Three different O_2_-tolerant [NiFe] hydrogenases from *Ralstonia eutropha* were found but they have lower enzyme activity than O_2_-sensitive hydrogenases [78]. Other O_2_-tolerant [NiFe] hydrogenases were also found in *Aquifex aeolicus*, *Escherichia coli*, and *Desulfovibrio fructosovorans* [79,80]. [NiFe] hydrogenase *Ko*Hyd3 purified from *Klebsiella oxytoca* HP1 displayed remarkable O_2_ tolerance and exhibited substantial hydrogen evolving activity under 10–20% O_2_ in the gas phase [81].

Studies on the O_2_-tolerant membrane-bound hydrogenase showed that O_2_ resistance originates from its unusual redox properties and kinetic behavior. The proximal iron–sulfur cluster located in [NiFe] hydrogenases contains an unusual [4Fe–3S]–6Cys cluster with two more cysteine residues compared to the standard [4Fe–4S]–4Cys cluster, which can transmit two electrons and may be responsible for regulating unusual redox potentials [82]. The mechanism of O_2_ tolerance is complex and inter-domain electron transfer between the distal clusters is proposed to be one way of increasing the O_2_ tolerance of [NiFe] hydrogenase [83]. In O_2_-tolerant [FeFe] hydrogenase, the O_2_ sensitivity of the H-cluster is strongly influenced by the protein environment and can be reversibly converted from the active state into the inactive state [77].

Limiting the diffusion of O_2_ to the active site is an alternative approach for O_2_ tolerance enhancement. Since the active site is located inside the [NiFe] hydrogenases and connected to the protein surface through a hydrophobic channel, the diffusion of O_2_ to the active site can be limited by reducing the gas channel size at the cavity interface with the active site. Two conserved hydrophobic residues (Val and Leu) are located at the end of the hydrophobic channel in O_2_-sensitive [NiFe] hydrogenases and they are replaced by larger Ile and Phe in O_2_-tolerant [NiFe] hydrogenases [84]. [FeFe] hydrogenase CpI was engineered in combination with cell-free mutant screening for improving O_2_ tolerance, and M4 mutant CpI^N160DI197VA280VN289D^ showed higher O_2_ tolerance than the wild-type CpI. After exposure to 1% O_2_ for 5 min, the wild-type CpI retained only 23% activity while the M4 mutant retained 62% [85]. Using rational mutant libraries of *Clostridium* [FeFe] hydrogenase, CpI^T356V/S357T^ was identified to be the most O_2_-tolerant variant and has an equivalent aerobic hydrogen production rate in the presence of 5% O_2_ [86]. Enzyme engineering was shown to be a feasible tool for upscaling biohydrogen production, and meanwhile, the cellular context is also considered to be of great importance [87].

### 4.2. Immobilization Technology

Hydrogenase has a high catalytic conversion rate and a low overpotential under mild conditions and has potential applications in replacing Pt as an electrocatalyst to develop hydrogen biofuel cells [88]. Immobilization technology can help hydrogenase to be reusable, maintain its stability and catalytic activity on the electrode surface, and improve electron-transfer efficiency [89].

The design of novel nanostructured electrodes for enzyme fixing can facilitate direct electron transfer between enzymes and solid carriers, thereby alleviating the demand for enzymes as electronic media and making biotechnological applications such as biofuel cells and biosensors simpler [88]. Covalent immobilization of [NiFe] hydrogenases onto SAM-modified gold surfaces makes enzymatic electrodes relatively stable, the rate of electron transfer increased, and redox mediators were not required [90].

Nanomaterials can be used to promote electron transfer efficiency. The study on the immobilization of Fd-HydA1 on black TiO_2_ nanotubes (bTNTs) found that direct electron transfer happened between black TiO_2_ and Fd-HydA1 [91]. The effect of molecular weight on the catalytic and electrochemical properties of hydrogenase was investigated by fixing truncated enzymes (*Pf*_αδ_ and *Pf*_α_, containing the subunits αδ and the α subunit only, respectively) derived from a four-subunit (αβγδ) [NiFe] hydrogenase *Pf*SHI to multiwalled carbon nanotubes (MWCNTs), and results showed that *Pf*_αδ_ with a shortened distance between the electrode and enzymes exhibited a higher electron transfer rate than *Pf*SHI [92].

### 4.3. Modification of Nitrogenase Substrate Selectivity

The electron flux through nitrogenases is largely independent of substrates being reduced, implying all nitrogenase substrates including N_2_ (for BNF), acetylene (to produce ethylene), and protons (for BHP) can compete for the same pool of electrons effectively [93].

Because hydrogen production by nitrogenases is independent of N_2_ reduction, the replacement of N_2_ by Ar can enhance the electron flux to proton reduction and keep away from N_2_ reduction. This method is effective to produce only H_2_, but the higher operational cost may follow [94].

Mutagenesis provides an alternative approach to overcome the N_2_ competition. The catalytic FeMo cofactor has been identified to provide substrate reduction sites, so a lot of research works had focused on its structure, its reactivity, and the development of genetic strategies for altering the substrate selectivity of dinitrogenase. Nonpolar or bulky residues such as Val, Phe, and Trp have functions in substrate access, substrate binding, and FeMo cofactor positioning. All of them constitute an important regulated network for enzymatic function, providing target sites in engineering [95].

Several valine substitutions in the α subunit of dinitrogenase have been intensively studied. The α-70Val site is predicted to have effects on the access of substrates to the active site. The substitution of V70A allows larger substrates, and V70I is just the opposite with the ability to block the access of acetylene and N_2_ to the active site except for protons [96,97]. Both hydrophilic and hydrophobic channels were supposed to be accessible for substrates to reach the buried active site. The α-71Val site conserved in Mo-nitrogenases is predicted to be in the hydrophobic channel and α-75Val is directly near the active site. Both α-71 and α-75 sites affect substrate specificity and modification of α-70Val, α-71Val, and α-75Val can result in higher hydrogen production [98,99].

### 4.4. Enzyme Compartmentalization

Bacterial microcompartments (BMCs) are found in a broad range of bacteria. They are self-assembled and functional analogs of eukaryotic organelles. A BMC is composed of an outer selectively permeable protein shell and an enzymatic core which performs a specific metabolic process and contributes to the functional diversity of BMCs [100]. BMCs can serve as physical barriers to protect cargo enzymes inside and provide a natural microenvironment for enhancing catalytic performance. BMCs have many potential applications for providing functional compartmentalization within cells to synthesize non-native metabolites or to deliver medical molecules [101].

Carboxysomes are anabolic BMCs and are found in all cyanobacteria and some chemoautotrophic bacteria. They have a proteinaceous icosahedral outer shell of roughly 800 to 1400 angstroms in diameter and they house enzymes involved in carbon fixation [102]. Carboxysome is prospective to be engineered for constructing nanoreactors. Carboxysome protein-encoding genes were expressed and self-assembled into robust carboxysome shells in *E. coli*. The empty shells were proved to have the capacity of encapsulating catalytic [FeFe] hydrogenases and functional partners together to create nanoreactors for hydrogen production and the O_2_ tolerance of the enzymes was improved at the same time [103].

### 4.5. Metabolic Engineering

Metabolic engineering provides a very promising strategy to improve hydrogen yield by redirecting biochemical pathways. Genetic engineering approaches have been used in both natural hydrogen-producing strains such as green microalgae and cyanobacteria and model organisms such as *E. coli* to favor hydrogen production. Genetically modified strains with a higher ability to generate biohydrogen have been successfully constructed [104,105]. Different modifications on hydrogenases and nitrogenases are summarized in Table 3.

### 4.6. Artificial Hydrogenases

**Figure 4:**
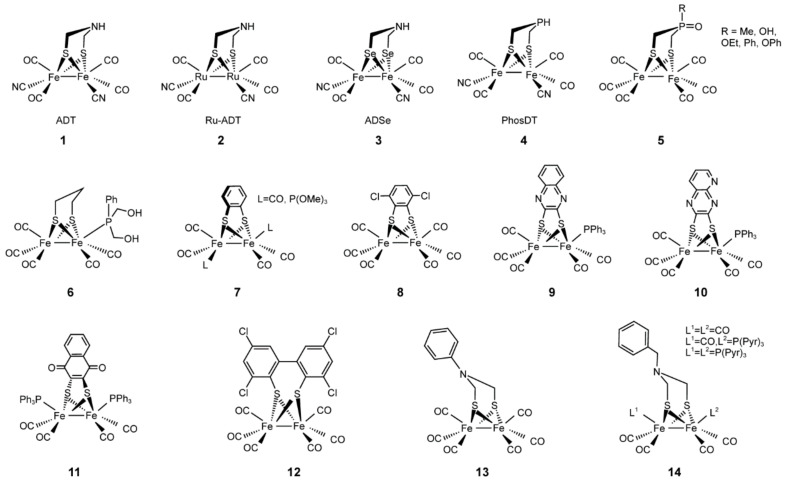

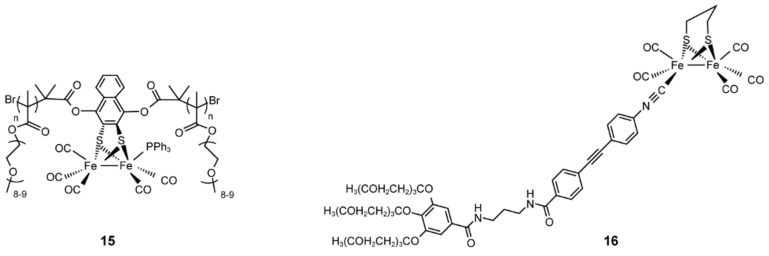
Various artificial hydrogenase mimics reported in the literature. References for these catalysts: **1**, [125]; **2**, [126]; **3**, [127]; **4** and **5**, [128]; **6**, [129]; **7**, [130]; **8**, [131]; **9** and **10**, [132]; **11**, [133]; **12**, [134]; **13**, [135]; **14**, [136]; **15**, [137]; **16**, [138].

Studies on molecular structures and catalytic mechanisms of diverse enzymes involved in biohydrogen production inspired researchers to develop novel catalysts as artificial hydrogenases and construct more stable and efficient catalytic systems for hydrogen gas generation (Figure 4).

The metal center substitution was used to prepare the first artificial hydrogenase nickel-substituted rubredoxin (NiRd) with a structure of a mononuclear Ni ion coordinated by four cysteine residues, and the artificial hydrogenase showed a capability of catalyzing hydrogen evolution [139]. In heme-binding proteins, the native cofactor iron protoporphyrin IX was replaced by cobaltous protoporphyrin IX (CoP) and the replacement resulted in modest catalysts for proton reduction to produce hydrogen. In addition, the CoP–myoglobin system showed strong O_2_-tolerant catalytic behavior [47]. Cobaloxime catalysts and electron transfer proteins with light-harvesting properties, such as ferredoxins and apo-flavodoxins, can efficiently self-assemble and provide the photocatalytic ability for proton reduction.

Many other efforts have been made to design various cofactors potentially to be integrated into [FeFe] hydrogenases. Both iron atoms were replaced with the non-native metallic element ruthenium and this [RuRu] analog of [FeFe] hydrogenase has the advantage to trap the key hydride intermediate state which is transient for [FeFe] hydrogenase. The stability of ruthenium hydrides can provide deep insight into the [FeFe] hydrogenase catalytic mechanism. In the analog, the catalysis reaction cannot proceed because the ruthenium atoms in the hydride intermediate state are redox-inactive [126]. In another study, the element nitrogen located at the bridgehead of the bridging dithiolate was substituted with its homolog phosphorous. Three new phosphorous-based [FeFe] hydrogenase mimics were synthesized by reacting (HSCH_2_)_2_P(O)R (R = Me, OEt, OPh) with Fe_3_(CO)_12_ and showed that the phosphorous could be reduced which may potentially improve catalytic activity regarding hydrogen evolution reaction [128]. The diiron carbonyl compounds with aromatic dithiolate bridges were also used to mimic the catalytic site of [FeFe] hydrogenase. They are robust and readily reducible, and aromatic dithiolate bridges are helpful for catalytic intermediate stabilization, molecular engineering, assembly with functional materials, and so on. These mimics exhibited relatively positive potentials as effective catalysts for electro- or photochemical hydrogen production [140].

The H-cluster of all [FeFe] hydrogenases consists of a cubane-like [4Fe–4S] cluster and a [2Fe–2S] cluster with additional CO and CN ligands and bridged by an azadithiolate (ADT) [128,141]. As the homogeneity and simplicity of the [2Fe–2S] sub-cluster and crystal structures of [FeFe] hydrogenases were determined, many synthetic cofactors were developed to replace the active site of [FeFe] hydrogenase and biohybrid systems were also investigated by using synthetic diiron carbonyl moieties and non-hydrogenase protein matrixes [142]. The synthetic [(μ-S_2_)Fe_2_(CO)_6_] motif can mimic the [2Fe] subsite of [FeFe] hydrogenases and provide evident photo-induced hydrogen production under photocatalytic conditions, which is valuable for designing noble metal-free catalysts for electrochemical hydrogen production. Due to the poor water solubility of [(μ-S_2_)Fe_2_(CO)_6_], a bioactivated [FeFe] hydrogenase mimic with two pyrene moieties was further prepared and integrated on multi-walled carbon nanotube (MWNT)-based electrodes through π-interactions to provide remarkable stability and activity in electrocatalytic hydrogen production under aqueous conditions [143]. The active site mimics also have potential applications for hydrogen fuel cells with more economical materials. Model compounds can be introduced into an environment to help them maintain catalytic effectiveness and stability. Successful examples include a covalent attachment to polymer backbones and oligopeptide chains, encapsulated in peptide hydrogels and micelles [130,144,145].

The “iron–sulfur world” theory proposes the FeS/H_2_S pair as the origin of life and a potential ancestor of [FeFe] hydrogenase [146,147,148]. Compartmentalization is considered to form an autocatalytic inorganic metabolic system to fulfill the requirements of life development. A membrane-bound [FeFe] hydrogenase model was prepared, and this vesicular system can exhibit catalytic action under particular conditions. This [FeFe] hydrogenase compartmentalization system can be applied as a minimal cell model or a nanoreactor to generate hydrogen [148].

Diiron complexes as mimic [FeFe] hydrogenase have been successfully immobilized on a metal–organic framework (MOF) or mesoporous silica (MS). Periodic mesoporous organosilica (PMO) with thiol groups (SH-PMO) was also developed for artificial [FeFe] hydrogenase anchoring for turnover number (TON) improvement [149]. 

Tremendous research works have been conducted to develop synthetic systems to mimic natural enzyme reactivity and even obtain enhanced stability and more remarkable catalytic activities. Undoubtedly, challenges to developing economical hydrogen production systems remain, and the efforts of understanding enzymes and their applications will continue.

## 5. Summary and Perspective

Due to the problems of environmental, energy, and sustainable development, more and more researchers and engineers are committed to developing biohydrogen production as a new alternative energy source. The core catalysts of biohydrogen production are two types of enzymes, namely, hydrogenase and nitrogenase, and each type of enzyme can be further divided into different groups with different structures and catalytic mechanisms. After decades of research, the structures and catalytic mechanisms of hydrogenases and nitrogenases have been well understood. The understanding of these mechanisms has led to many studies on the protein engineering of hydrogenase and nitrogenase. At the same time, due to the wide distribution of hydrogenase and nitrogenase in nature, many new hydrogen-producing catalysts with excellent properties have been discovered in the natural environment. The research on hydrogenase and nitrogenase also provides new ideas for the development of artificial enzyme-mimic catalysts. Artificial hydrogenases have been developed and widely studied.

However, due to the complex structure of these two enzymes, especially the complex metal clusters, it is very challenging to design and engineer them rationally. Revealing the structural and functional relationship of these two enzymes, especially the allosteric effect of distal residues on function, is still the focus of research in the future. At the same time, only a small number of enzymes have been studied in detail, while the structure and function of a large number of hydrogenases and nitrogenases in different genome-sequenced species are still limited, which may be important research targets in the future. As more data on the relationship between enzyme structure and activity are accumulated, it will be possible to better design modification sites and engineering protocols for new enzymes, further improving their efficiency, O_2_ tolerance, and other properties.

The efficiency of biohydrogen production is not only determined by the efficiency of the core catalysts but it is also affected by microbial metabolism, which, in turn, is complicated by the substrate types, fermentation process, reactor design, etc. Therefore, to achieve the economic feasibility of biohydrogen production, it is necessary to comprehensively consider all aspects from the enzyme to the process, to realize the improvement of hydrogen production efficiency and the ultimate technical and economic feasibility. Among them, microbial metabolic engineering is the core and key to adapting these enzymes to different substrate types, especially complex waste materials. Furthermore, combined with waste treatment, DFBHP is the most promising strategy to obtain economical and sustainable biohydrogen production. Due to the limited efficiency and scale of hydrogen production by microorganisms, the development of artificial enzyme-mimic catalysts based on the catalytic principles of hydrogenase and nitrogenase has been a rapidly developing field in recent years. Since chemical catalysts are no longer limited to the growth and metabolic rate and scale of biological systems, they have good prospects for large-scale scaling up. Nevertheless, the development of a sustainable, green, and efficient biohydrogen production process is one of the important solutions to solve energy and environmental problems in the future, and the realization of this requires continuous research and innovation on the basic science and technology issues in biohydrogen production.

## Figures and Tables

**Table 2:** Comparison of enzyme O_2_ sensitivity and hydrogen production rate [17].

Enzymes	O_2_ Sensitivity	Hydrogen Production Rate
Nitrogenases	Significantly sensitive	3–4 fold lower than [FeFe] hydrogenases
[NiFe] hydrogenases	Inactivated reversibly	100-fold lower than [FeFe] hydrogenases
[FeFe] hydrogenases	Highly sensitive and inactivated irreversibly	Highest
[Fe] hydrogenases	Resistance to O_2_	—

**Table 3:** Strategies for hydrogenases and nitrogenases metabolic engineering.

Strategies	Metabolic Pathways	Methods and Genes	Effects	Organisms	Reference
Reducing hydrogen consumption	Central carbon metabolism	Deletion of *hya* and *hyb*	Disrupting uptake hydrogenase	*E. coli*	[106,107]
	Fermentative hydrogen production	Disruption of *hyh* and *alaAT*	Inactivating two hydrogen consumption enzymes	*Thermococcus kodakarensis*	[108]
	Fermentative hydrogen production	Cloning *hoxEFUYH* from the cyanobacterium	Inhibition of hydrogen uptake activity	*E. coli*	[109]
	Electron transfer step	Mutation of C12P in fusion protein f-HupS	Modification of Fe–S cluster in uptake hydrogenase	*Nostoc punctiforme* ATCC 29133	[110]
	Consumption of hydrogen by uptake hydrogenase	Site-directed mutagenesis	Disruption of uptake hydrogenase	*Rhodobacter sphaeroides* O.U.001	[111]
Improving hydrogen-producing enzymes	Fermentative hydrogen production	Using a stronger constitutive promoter to replace the promoter of membrane-bound [NiFe]-hydrogenase	Overexpressing [NiFe] hydrogenase	*Thermococcus kodakarensis*	[108]
	Fermentative hydrogen production	Insertion of *hydACa* and *hydACb*	Overexpressing two [FeFe] hydrogenases	*Clostridium acetobutylicum* DSM 792	[112]
	Anaerobic dark fermentation	Cloning multiple copies of *hydA*	Overexpressing [Fe] hydrogenase	*Clostridium paraputrificum*	[113]
	Electron transfer step	Mutation of R171D in HydA1	Enhancing [FeFe] hydrogenase catalytic activity	*Chlamydomonas reinhardtii*	[114]
	Electron flow	Cloning *rnf* operon	Overexpressing the Rnf complex to increase the supply of reductants	*Rhodobacter sphaeroides* 2.4.1	[115]
	Electron flow	Cloning *rnf* operon under different promoters	Overexpressing Rnf complex, enhancing nitrogenase activity	*Rhodobacter capsulatus* SB 1003	[116]
	Electron transfer flux	Insertion of *fdxN*	Overexpressing fdxN (electron donor), enhancing nitrogenase activity	*Rhodobacter sphaeroides* HY01	[117]
	Photo-fermentative hydrogen production	Mutation of nitrogenase-regulating genes	Enhancing nitrogenase activity	*Rhodopseudomonas palustris*; *Rhodobacter sphaeroides* HY01; *Rhodobacter sphaeroide*s	[118,119,120]
Gene coexpression	Photoheterotrophic hydrogen production	Cloning fermentative metabolic genes including [Fe] hydrogenase	Expression of FHL, [Fe] hydrogenase, and nitrogenase	*Rhodobacter sphaeroides* KCTC 12085	[121]
Redirecting metabolic pathways	Dark fermentative hydrogen production	Construction of synthetic pyruvate:H_2_ pathway	Co-expression of six proteins including [FeFe]-hydrogenase	*E. coli* BL21	[122]
	Redox balancing pathway	Deletion of uptake hydrogenase gene	Inactivation of Calvin–Bensone–Bassham (CBB) pathway	*Rhodobacter capsulatus* YO	[123]
	Electron flow, ammonia tolerance	Mutation of *hupSL*, *phbC*, *pucBA*	Elimination of nonessential reductive pathways	*Rhodobacter sphaeroides* 2.4.1	[115]
Reducing gas tolerance	Aerobic fermentative hydrogen production	Cloning *hydS* and *hydL* from *Hydrogenovibrio marinus*	Heterologous expression of O_2_-tolerant [NiFe]-hydrogenase	*E. coli*	[124]
	Ammonia tolerance	Mutation of *nifA*	Expression of ammonia-tolerant NifA	*Rhodobacter sphaeroides* 2.4.1	[115]
	photo-fermentative hydrogen production	Mutation of nitrogenase-regulating genes	Ammonium tolerance improvement	*Rhodopseudomonas palustris*; *Rhodobacter sphaeroides* HY01; *Rhodobacter sphaeroides*	[118,119,120]

## Data Availability

No new data were created or analyzed in this study. Data sharing is not applicable to this article.

## Sample Availability

Samples of the compounds are not available from the authors.
